# Effect of BoNT/A in the Surface Electromyographic Characteristics of the Pelvic Floor Muscles for the Treatment of Chronic Pelvic Pain

**DOI:** 10.3390/s21144668

**Published:** 2021-07-07

**Authors:** Monica Albaladejo-Belmonte, Francisco J. Nohales-Alfonso, Marta Tarazona-Motes, Maria De-Arriba, Jose Alberola-Rubio, Javier Garcia-Casado

**Affiliations:** 1 Centro de Investigación e Innovación en Bioingeniería, Universitat Politècnica de València, 46022 Valencia, Spain; moalbel@ci2b.upv.es; 2 Servicio de Ginecología y Obstetricia, Hospital Politècnic i Universitari La Fe, 46026 Valencia, Spain; franoal@uv.es (F.J.N.-A.); tarazona_marmot@gva.es (M.T.-M.); dearriba_margar@gva.es (M.D.-A.); 3 Unidad de Bioelectrónica, Procesamiento de señales y Algoritmia, Instituto de Investigación Sanitaria La Fe, 46026 Valencia, Spain; pepe_alberola@iislafe.es

**Keywords:** chronic pelvic pain, botulinum toxin, pelvic floor muscles, surface electromyography

## Abstract

Chronic pelvic pain (CPP) is a complex condition with a high economic and social burden. Although it is usually treated with botulinum neurotoxin type A (BoNT/A) injected into the pelvic floor muscles (PFM), its effect on their electrophysiological condition is unknown. In this study, 24 CPP patients were treated with BoNT/A. Surface electromyographic signals (sEMG) were recorded at Weeks 0 (infiltration), 8, 12 and 24 from the infiltrated, non-infiltrated, upper and lower PFM. The sEMG of 24 healthy women was also recorded for comparison. Four parameters were computed: root mean square (*RMS*), median frequency (*MDF*), Dimitrov’s index (*DI*) and sample entropy (*SampEn*). An index of pelvic electrophysiological impairment (*IPEI*) was also defined with respect to the healthy condition. Before treatment, the CPP and healthy parameters of almost all PFM sides were significantly different. Post-treatment, there was a significant reduction in power (<*RMS*), a shift towards higher frequencies (>*MDF*), lower fatigue index (<*DI*) and increased information complexity (>*SampEn*) in all sites in patients, mainly during PFM contractions, which brought their electrophysiological condition closer to that of healthy women (<*IPEI*). sEMG can be used to assess the PFM electrophysiological condition of CPP patients and the effects of therapies such as BoNT/A infiltration.

## 1. Introduction

Chronic pelvic pain (CPP) syndrome is defined as a persistent or recurrent pelvic pain that lasts more than 6 months and has no proven infection or obvious pathology to account for its presence [1]. It can involve different organ systems such as the sexual, musculoskeletal, neurological or psychological and can be associated with a vast number of different symptoms, often in the form of dyspareunia (pain during intercourse) and vulvodynia (vulvar pain) in female patients [2].

Due to CPP’s complex nature, there is little information on its socioeconomic impact. Some studies estimate that CPP affects between 5.7% and 26.6% of the population, mostly in fertile women [3], and that cases with vulvodynia as the main symptom involve an economic burden of between USD 31 and 72 billion per year in the US [4]. Its impact on patient’s lives can disturb their professional and sexual life, sleep quality, relationships and self-perception, as well as the ability to perform physical activities and housework [5]. CPP is difficult to manage effectively in many cases, so its persistence can lead patients to develop feelings of isolation, invalidation and anxiety [6].

Myofascial pelvic pain syndrome, i.e., pain in the muscles or connecting fascia of the pelvic floor, plays a leading role in many CPP cases [7]. Some authors estimate that more than 85% of cases have a pelvic floor muscle (PFM) dysfunction [8], suggesting that surface electromyography (sEMG) could be used for its diagnosis and evaluation [9]. Probes containing surface electrodes inserted in the vaginal/anal opening are typically employed to record the activity of the PFM [10]. However, they can be uncomfortable for the patient, they are vulnerable to motion artifacts and their signal is sensitive to factors such as vaginal temperature or moisture [11].

Botulinum neurotoxin type A (BoNT/A) infiltration in the PFM is one of the most popular treatments, especially in cases where the muscle is largely responsible for the pain [8]. In this process, the toxin binds to the terminals of alpha presynaptic motoneurons and prevents them from releasing acetylcholine into the synaptic cleft. Neuromuscular synapsis is thus inhibited and the muscle fibers become unable to develop action potentials to contract. This weakens the muscles, and the effect is at its maximum in less than 2 weeks and lasts from 3 to 6 months [12,13,14]. BoNT/A also blocks the release of some nociceptive neuropeptides, which have a positive feedback on the genesis of inflammation and pain [12]. Different authors have studied and proved BoNT/A’s effectiveness in alleviating multiple symptoms associated with CPP [15].

The effectiveness of this treatment has traditionally been assessed from a clinical perspective of the improvement of painful symptoms and quality of life. Even though its effect on PFM contractility has also been monitored in some studies, the assessment relies on a subjective physical examination and does not always include vaginal pressure [16,17]. No previous work has fully described changes in the PFM’s electrophysiological condition after BoNT/A infiltration.

The aims of the present work were: (1) to study the changes in the PFM electrophysiological state of women with CPP associated with myofascial pelvic syndrome after treatment with BoNT/A, (2) to determine whether these changes lead to a more or less non-pathological electrophysiological state and (3) to define an indicator to assess the degree of PFM electrophysiological impairment and assist in the patient’s clinical evaluation. The sEMG of the PFM of both CPP patients and healthy women was recorded by adhesive electrodes. Features related to the energy, spectral content and complexity of the signal on different PFM sides were computed during the voluntary contraction and relaxation of the PFM in patients before BoNT/A infiltration and at different weeks after. A global index of pelvic electrophysiological impairment with respect to the healthy condition was also defined and computed. The results obtained show that significant changes take place in patients’ PFM activity after their treatment, which improves their electrophysiological status and alleviates pain.

## 2. Materials and Methods

### 2.1. Clinical Trial Overview and Sample Description

This study is part of a prospective, minimally invasive, non-masked and non-randomized phase III clinical trial performed at the Hospital Universitari i Politècnic La Fe (Valencia, Spain): “Electromyographic Study for the Help and Guidance of Botulinum neurotoxin A Administration in the Treatment of Chronic Pelvic Floor Pain (SEMG)” (Clinical Trials: NCT03715777). It meets the Declaration of Helsinki and was approved by the ethics committee of the hospital in July 2017. Women were recruited who presented CPP for more than 6 months, whose main manifestation was dyspareunia associated with a myofascial syndrome. Additional inclusion criteria were: 18 years old or older, no active pelvic infections, no general malignant, pelvic or psychiatric diseases, no contraindication to BoNT/A infiltration and no experimental drug administered 30 days before the beginning of the clinical trial. Twenty-five patients enrolled in the study, although one of them was lost in the follow-up. Twenty-four women with similar demographic and obstetric characteristics to those of the patients and with no pelvic conditions were also recruited. Both patients and healthy subjects gave their informed consent after the nature of the study was explained to them.

Table 1 shows a summary of the most relevant demographic and obstetric characteristics of the patients and healthy subjects, as well as some other aspects related to CPP in the case of the patients. There were no significant differences between the age, weight, height, nº of deliveries (Wilcoxon rank-sum test, confidence level: 5%) or menopause prevalence (Fisher’s exact test, confidence level: 5%) in both groups. In patients, pain was mostly associated with the left side (41.7%) of the pelvic floor or was bilateral (41.7%). A remarkable number of patients (66.7%) presented hypertonic PFM at the onset of the study.

Five medical visits were scheduled by the Gynecology and Obstetrics Service of the hospital for each patient, whereas healthy subjects were given a single visit. Patients attended a preliminary appointment before the study, when anamnesis and a physical exploration were performed; a visit at Week 0, when BoNT/A was infiltrated; and three more follow-up consultations at Weeks 8, 12 and 24. Their painful symptoms, sexual function and quality of life were evaluated at the beginning of the study and at Weeks 12 and 24 by different clinical questionnaires. Reduction of pain, quantified with the Visual Analogue Scale (VAS) of 11 points (0–10) [18], was the main clinical outcome assessed. VAS values significantly diminished from 7.0 ± 1.2 before BoNT/A treatment to 2.8 ± 2.4 and 2.3 ± 1.8 at Weeks 12 and 24, respectively, showing a reduction in painful symptoms. All the patients reported a lower value at both weeks, except for one patient at Week 12.

### 2.2. BoNT/A Infiltration

A gynecologist examined the contractile state and the sensitivity of the patient’s superficial and deep PFM areas by digital palpation. Ninety units of incobotulinumtoxinA (Xeomin^®^, Merz Pharmaceuticals GmbH, Frankfurt, Germany) were then diluted in 2 mL of lidocaine at 2% and injected by needle at a single point in the pubococcygeus muscle below the vaginal opening and above the anus. Injection was on the side with predominant pain; 18 patients on the left PFM and 6 on the right, followed by a massage of the zone.

### 2.3. Signal Acquisition

**Figure 1:**
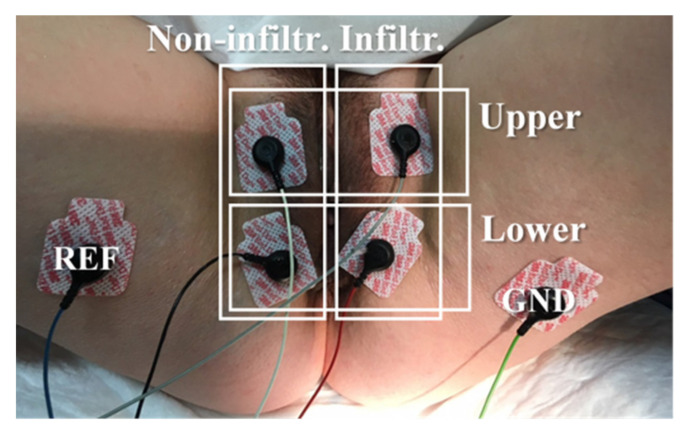
Electrodes arrangement for the acquisition of the bipolar sEMG of the infiltrated, non-infiltrated, upper and lower sides of the PFM.

The sEMG of the PFM of patients and healthy subjects was acquired by disposable Ag/AgCl electrodes (Red Dot 2660-5, 3M, St. Paul, MN, USA): four on the labia majora, a reference electrode (REF) and a ground electrode (GND) on the iliac spines. Four different bipolar signals were recorded, two from each vertical region (infiltrated and non-infiltrated in patients, left and right in healthy subjects) and two from each horizontal area (upper, lower), as depicted in Figure 1. The skin was previously carefully exfoliated with an abrasive gel (Nuprep 114g, Weaver and Company, Aurora, CO, USA) to reduce skin-electrode impedance.

Bipolar recordings were amplified with a multipurpose amplifier (Grass 15LT+4 Grass 15A94, Grass Instruments, West Warwick, RI, USA), which filtered them between 3 Hz and 1000 Hz. Signals were digitalized at a sampling rate of 10 kHz with a 16-bit analog-to-digital converter.

During signal recording, the subjects remained in a dorsal lithotomy position. After 3 min of basal recordings in a relaxed state, they were asked to perform a protocol of muscular activation. They carried out five maximum voluntary tonic contractions of 5 s spaced with relaxing periods of 10 s. Before starting the protocol of voluntary contractions, the physician explained to the patient that the performance of a maximum contraction was equivalent to holding urine at a maximum effort, asked her to carry out some contractions to check that she executed them correctly and provided feedback when she did not. During the protocol, the physician used the verbal instructions “contract” and “relax” to indicate the start and end of each contraction, respectively.

In the case of patients, the protocol and sEMG acquisition were carried out at Weeks 0, 8, 12 and 24. Signals’ preprocessing, characterization and statistical analysis were performed with the software MATLAB studio (version 2018b).

### 2.4. Signal Preprocessing and Annotation

Bipolar sEMGs were digitally filtered. First, 17th order high-pass (30 Hz cut-off frequency) and 8th order low-pass (450 Hz cut-off frequency) Butterworth filters were used to remove frequency components outside the frequency range of the PFM sEMG components. Cut-off frequencies were similar to those previously chosen by other authors in works also focused on the analysis of PFM sEMG [19]. Secondly, a comb filter (quality factor = 30) was used to eliminate the power interference (50 Hz) in the signal. All these filters were applied symmetrically to avoid introducing a lag in the signal.

Five intervals of contractile activity of approximately 5 s and one interval of basal activity of 10 s were manually annotated, avoiding possible signal artifacts, in the sEMG of each subject and recording session. The basal interval was selected prior to the first contraction with the PFM in a state of minimal activity [20].

### 2.5. Signal Characterization

To characterize the PFM myoelectrical activity in states of contraction and relaxation, four parameters were computed from preprocessed sEMG segments. Results from the 5 contractile segments of each recording session were averaged to obtain a single characteristic value of this condition. The computed parameters were:

1. *Root mean square (RMS)*. Given a discrete time series *x*[*n*] of *N* samples, its *RMS* is calculated according to the following formula:
  (1) $$RMS=\sqrt{\frac{1}{N}\sum_{k=1}^{N}x{\left[k\right]}^{2}}$$
  As this is a measure of signal power, it can be used to characterize the motor units’ level of physiological activity during PFM contraction [21].
2. *Median frequency (MDF)*. Considering a discrete time series sampled at a frequency *f_s_* and its power spectrum density (PSD), *MDF* can be formulated as follows:
  (2) $$\sum_{j=0}^{MDF}PSD\left(j\right)=\sum_{j=MDF}^{f_{s}/2}PSD\left(j\right)=\frac{1}{2}\sum_{j=0}^{f_{s}/2}PSD\left(j\right)$$
  where *PSD(j)* is the value of the PSD at frequency *j*. *MDF* is the frequency that divides the PSD into two regions with the same total energy [22]. Its value is highly dependent on the conduction velocity of the muscle fibers’ action potential, the waveform of the intracellular action potential and the volume conductor above the muscle [23,24].
3. *Dimitrov’s Index (DI)*, defined as follows [25]:
  (3) $$DI=\left(\sum_{j=0}^{f_{s}/2}j^{-1}\cdot PSD\left(j\right)\right)/\left(\sum_{j=0}^{f_{s}/2}j^{k}\cdot PSD\left(j\right)\right)$$
  where *k* is a positive number higher than the unit. Here, *k* = 5 was chosen, since this is the value commonly used in the literature [26].
  As *MDF*, *DI* characterizes the spectral content of the sEMG. It better quantifies peripherical muscle fatigue than conventional indexes [27]. An increase of its value may be associated with the development of this condition [25].
4. *Sample entropy (SampEn)*. This is the negative of the natural logarithm of the conditional probability that two numerical series of *N* samples that are similar at *m* points (probability:$\;B^{m}\left(r\right)$) keep being similar at the following point (probability: $A^{m}\left(r\right)$), ignoring self-matches and considering *r* as the dissimilarity tolerance [28]:
  (4) $$SampEn\left(N,\;m,\;r\right)=-\log\frac{A^{m}\left(r\right)}{B^{m}\left(r\right)}$$

Further details of the computation of $A^{m}\left(r\right)$ and $B^{m}\left(r\right)$ are available in [28]. In this study, signals were normalized to have zero mean and unit standard deviation before computation. The parameters *m* = 2 and *r* = 0.15 were set following recommendations in the literature [29]. As *SampEn* informs on the rate of new information generation, it can be regarded as a measure of the level of complexity or irregularity of the PFM sEMG, so that a greater value implies a higher level of signal complexity [28].

The index of pelvic electrophysiological impairment (*IPEI*), which considers the deviances with respect to the healthy condition of all sEMG parameters at each recording site, was defined and computed for each patient as follows:

(5) $$IPEI=\frac{\sum_{i=1}^{M}{\left[\left(y_{i}-\bar{X_{i}}\right)/\;\sigma_{i}\right]}^{2}}{M}$$

where $y_{i}\;$ is the value of the *i^th^* sEMG feature in the patient; $\bar{\;X_{i}}$ and $\sigma_{i}$ are the mean and the standard deviation of that feature in the healthy group, respectively; and *M* is the total number of features assessed (4 parameters). In other words, *IPEI* is the average normalized Euclidean distance between the sEMG of each patient and those of healthy subjects, considering the information of all the parameters. *IPEI* was also computed for each healthy subject as an intraclass measure.

### 2.6. Data Analysis

Normality of the data was evaluated according to the Kolmogorov–Smirnov test (significance level: 5%). Two types of statistical tests were applied to assess significant differences in the sEMG parameters:

(1) Test 1: evolution of the patients’ PFM activity over the weeks of the study. For a given parameter, state of PFM activation (contraction/relaxation) and bipolar channel, to check whether there were statistical differences between patients’ datasets at Week 0 vs. Week i, with i = {8, 12, 24}. The samples of the datasets compared in each test were paired, i.e., the value computed for a patient at a given week was compared with the one obtained for the same patient in a different week. As the datasets did not come from a normal distribution, Test 1 was a two-tailed Wilcoxon signed-rank test under a confidence level of 0.05.
(2) Test 2: comparison of the PFM activity of patients and healthy subjects. For a given parameter, state of PFM activation, bipolar channel and week of the study, to check whether there were statistical differences between the datasets of patients and healthy women. For each pair of datasets, which followed a non-normal distribution, a two-tailed Wilcoxon rank-sum test was performed under a 0.05 confidence level. In the case of vertical channels, since BoNT/A was infiltrated on the left side in most cases, comparisons were: patients’ infiltrated side vs. healthy subjects’ left side and patients’ non-infiltrated side vs. healthy subjects’ right side.

## 3. Results

**Figure 2:**
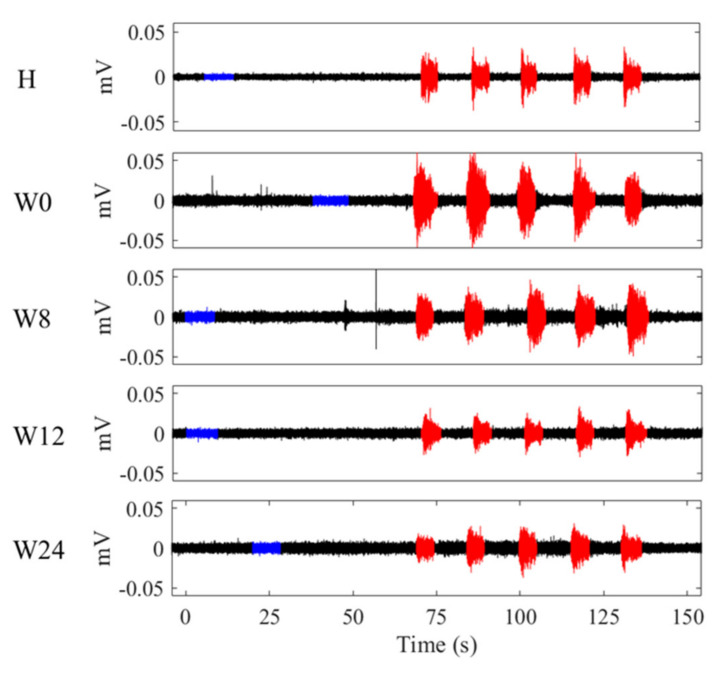
sEMG recorded from the lower side of the PFM of the #9 healthy subject (H) and of the #7 patient throughout the weeks of the study (W0, W8, W12, W24). (Red: annotated contractions. Blue: annotated basal segment before contractions).

Figure 2 shows the sEMG acquired from the lower side of the PFM of a CPP patient each week (W) of the clinical trial, as well as that of a healthy subject (H). Annotated contractile segments are highlighted in red, while basal segments are blue. It can be seen that the amplitude of the patient’s sEMG diminished as the study progressed, especially during the PFM contraction, and that it was greater than the amplitude of the healthy woman’s sEMG.

**Figure 3:**
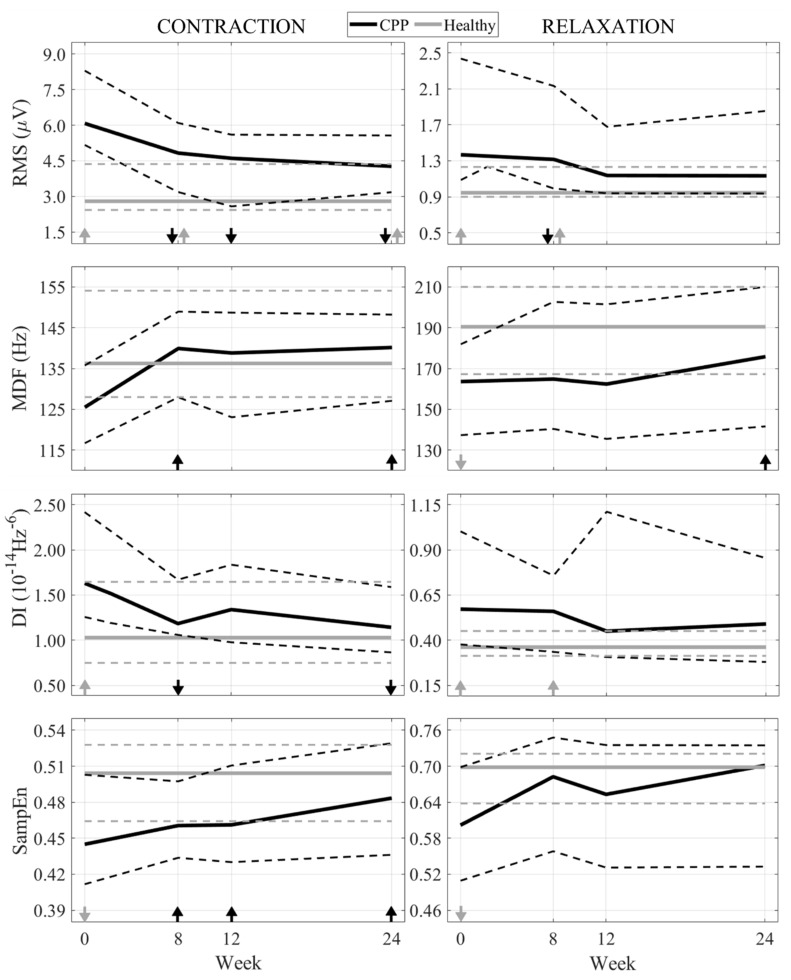
Evolution of RMS, MDF, DI and SampEn during PFM contraction (**left**) and relaxation (**right**) of CPP patients (black lines) and healthy subjects (grey lines) throughout the study. Their medians (solid line) and 25 and 75 percentiles (broken lines) are displayed. Statistically significant differences (*p* < 0.05) between Week 0 and subsequent weeks (Test 1) are shown with a black arrow (downwards if the parameter decreases, upwards if it increases) and between patients and healthy subjects at a given week (Test 2) with a grey arrow (downwards if the parameter is lower in patients than in healthy women, upwards if it is greater).

Figure 3 depicts each parameter computed from the sEMG signals of the patients’ infiltrated side (black lines) each week of the study and of the left side of healthy subjects (grey lines). Their medians are plotted with solid lines and their 25 and 75 percentiles with broken lines. Subplots on the left show the results obtained during PFM contractions, while those on the right give the values at rest. Arrows over their *x*-axis show statistically significant differences, pointing upwards or downwards depending on whether the parameter increased or decreased its value, respectively, with regard to Week 0 (Test 1, black arrows) or to the healthy condition (Test 2, grey arrows).

Figure 3 shows that the *RMS* of the signal recorded from the infiltrated side decreased after BoNT/A infiltrations. During PFM contractions, significantly lower values were obtained at Weeks 8, 12 and 24, while they were only observed at Week 8 when segments of basal activity were analyzed. Values of *MDF* and *DI* increased and decreased, respectively, in the weeks after the treatment, with statistically significant differences in contractions at Weeks 8 and 24. The *SampEn* of the signal rose after infiltration, significantly during PFM contractions in all follow-up sessions. It can be seen in Figure 3 that all parameters were significantly different between the patients and healthy subjects at the beginning of the study, except for the *MDF* during PFM contractions. After BoNT/A infiltration, these changes in the patients’ sEMG brought their parameters closer to those of their healthy counterparts. Figure 3 shows that significant differences between both groups disappeared at the end of the study, or even some weeks before, in all cases except for the *RMS* during contractions.

Table 2 and Table 3 show the medians and interquartile ranges of parameters computed from signals of all channels during PFM contractions and relaxation, respectively. Table 4 and Table 5 show a summary on the results provided by Tests 1 and 2, respectively. Significant differences are indicated by shaded cells. Up and down arrows indicate patient parameter values significantly greater or smaller than before BoNT/A treatment (Table 4) and those of healthy women (Table 5).

As Table 2 shows, *RMS* decreased during contractions from Week 0 to Weeks 8, 12 and 24, reaching its minimum value at Week 24 in the infiltrated, non-infiltrated and upper sides and at Week 12 in the lower side. According to Table 4, differences between Week 0 and each follow-up visit were significant in all sides, except for the non-infiltrated side at Week 12. During PFM relaxation, the *RMS* showed a decreasing trend with lower values than before the treatment in all sides. As in the contractions, the minimum was reached at Week 12 in the lower side and at Week 24 in the rest. However, Table 4 shows that these differences with respect to Week 0 were significant only in some occasional weeks and channels: at Week 8 in the infiltrated side and at Week 24 in the non-infiltrated and lower sides.

According to Table 2 and Table 3, *MDF* increased in the lower side during PFM contraction and relaxation throughout the study, with significant differences at Weeks 8 and 24 in the case of contractions and also at Week 12 during relaxations (Table 4). The non-infiltrated and upper sides also showed increasing trends but were interrupted by a drop at Week 12 to non-significantly lower values than before BoNT/A infiltration. *MDF* reached the maximum value at Week 24 in all channels and during both states of PFM activation, except for the lower side at PFM rest. Conversely, *DI* tends to decrease in the follow-up sessions for all sides during contractions and relaxations, with minimum values at Week 24, except for the infiltrated side during relaxation, whose median *DI* was lower at Week 12. Reductions of *DI* during PFM contractions were significant at Weeks 8 and 24 with respect to Week 0 in all sides, except for the upper side at Week 8 (Table 4). During PFM rest, differences were significant at Weeks 8 and 24 only in the lower side. The *SampEn* value experienced a slight rise throughout the weeks after the treatment during contractions and relaxation. All sides reached their maximum values at Week 24, except for the upper side during rest at Week 8. During PFM contractions, they were significantly higher than before BoNT/A treatment in all channels but the non-infiltrated one.

Regarding the results provided by Test 2, it can be seen in Table 5 that there were significant differences between patients and healthy women at Week 0 for almost all parameters during contraction and relaxation, not only in the infiltrated (most painful) side but in all sides in most cases, with a lower incidence in the upper and lower sides. These significant differences disappeared after treatment at Week 24 or even before. The only exceptions were the *RMS* values of the infiltrated side during PFM contractions and of the non-infiltrated side at both states of activation, as well as the *SampEn* of this side during contractions. In these cases, although significant differences were still found between both groups at Week 24, Table 2 and Table 3 show that the values from patients and healthy women were more similar than at the beginning of the study (Week 0).

**Figure 4:**
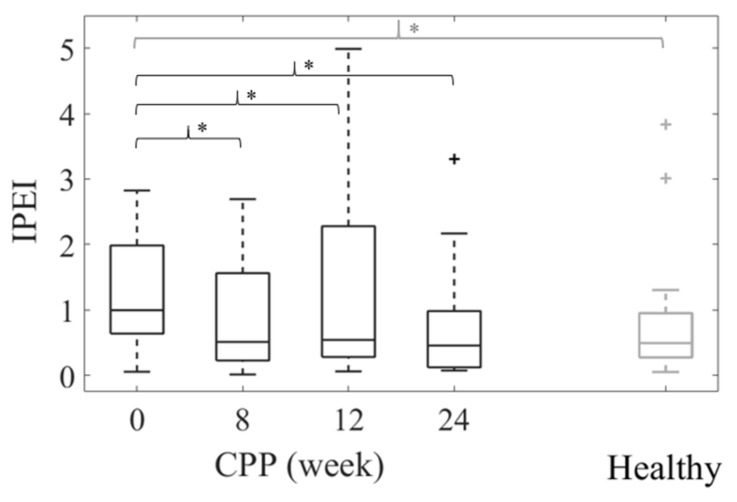
Box-whisker plots of *IPEI* values during PFM contractions of the infiltrated side of CPP patients throughout the study (black boxes) and of the left side of healthy subjects (grey boxes). Statistically significant differences (*p* < 0.05) between Week 0 and subsequent weeks (Test 1) are shown with a black brace and asterisk and between patients and healthy subjects at a given week (Test 2) with a grey brace and asterisk.

Figure 4 shows the *IPEI* values computed from the sEMG features of the infiltrated side of patients during PFM contractions, for which a greater effect of the drug was found (Table 4), and of the left side of healthy subjects. Differences between the patients’ *IPEI* each week with respect to Week 0 (Test 1, black braces) and with respect to the healthy group’s *IPEI* (Test 2, grey braces) are shown. The median of patient’s *IPEI* decreased from Week 0 to Weeks 8, 12 and 24, with significant differences in all follow-up visits. When it was compared with the healthy subjects’ *IPEI*, significant differences were obtained between both groups at Week 0, but they disappeared after treatment. Figure 4 also shows that the interquartile range of the patients’ *IPEI* was notably larger than that of the healthy group even at Weeks 8 and 12, but both became almost identical at the end of the study.

## 4. Discussion

In this study, the PFM electrophysiological condition of female patients with CPP mainly associated with a myofascial syndrome was characterized both before and after their treatment with BoNT/A, as well as that of healthy women with similar demographic and obstetric characteristics. For this, the sEMG of their PFM was recorded using adhesive electrodes on the vulva, and their energy, spectral content and complexity were assessed according to four parameters: *RMS*, *MDF*, *DI* and *SampEn*. Significant reductions of *RMS* and *DI* and increments of *MDF* and *SampEn* were obtained after the infiltration, mainly during the PFM contraction, at Weeks 8, 12 and 24.

Before BoNT/A treatment, *RMS* values computed during the contraction and relaxation of the PFM were significantly higher in the vertical sides of the PFM of pathological subjects than in those of healthy women. This result indicates that the recruitment and/or discharge rates of the motor units of the PFM was significantly increased in patients. This was an expected outcome, since muscle hyperactivity is a characteristic feature of chronic pelvic pain syndromes in which the PFM has the main role [30]. After BoNT/A treatment, *RMS* values of patients significantly decreased with respect to those calculated at the beginning of the study, becoming more similar to their healthy counterparts. This drop in the power of the patients’ PFM activity would be a consequence of the chemical denervation of some motor units after BoNT/A treatment, which prevented them from being recruited. The evaluation of the sEMG signal after BoNT/A infiltration has not been previously addressed in patients with CPP associated with a myofascial syndrome. However, considering that the myoelectrical activity of a muscle is the cause of its mechanical activity [31], we can conclude that our results are consistent with those obtained by Morrissey et al. [32], who monitored the intravaginal pressure of women with refractory high-tone pelvic floor dysfunction before and after BoNT/A treatment, reporting a reduction during PFM contractions and relaxations 4, 8, 12 and 24 weeks after infiltration.

Before treatment, patients showed significantly lower *MDF* values than healthy women. After BoNT/A infiltration, these values increased, becoming more similar to those of their healthy counterparts. This rise in patients’ *MDF* values implies that the PSD of their sEMG shifted towards higher frequencies after the treatment. Muscle fatigue would justify not only why patients showed higher *MDF* values over the follow-up but also why they were significantly lower than those of healthy women before BoNT/A infiltration. The fatigue index of a muscle is higher in hypertonic or spastic conditions, as could be the case of CPP patients with myofascial syndrome suffering a longstanding overactivation of the muscle, than in a physiological state [33]. Fatigued muscles are characterized by a reduced conduction velocity of their fiber muscles and an increased synchronization of motor units, which causes a shift in the PSD of their sEMG towards lower frequencies [34]. The results obtained in this study thus suggest that the chemical denervation induced by BoNT/A led to PFM recovery from a previous state of fatigue. This interpretation is reinforced by the fact that *RMS* values were also reduced after BoNT/A infiltration. As described in [24], a reduction of muscle fatigue is associated with a reduced amplitude and a higher *MDF*. In contrast to us, Kim et al. [35] reported a reduction of the signals’ MDF after infiltrating BoNT/A into the masseter of 19 healthy women that developed pain and muscle fatigue after performing a sustained clench. They treated a structure completely different to the PFM, from an anatomical and electrophysiological point of view, and whose pathological behavior was not associated with a chronic clinical disorder such as CPP but with an occasional muscle overstrain. Moreover, they registered electromyographic signals with needle electrodes; therefore, all these differences could account for the discrepancies between our results and theirs. *DI* was computed to complement and strengthen the assessment of PFM fatigue throughout the clinical trial. A lower value was obtained in patients at Weeks 8, 12 and 24 (with regard to Week 0). This outcome strengthens the hypothesis of the PFM recovery from fatigue, since a higher *DI* is associated with the development of peripheral muscle fatigue [27].

The complexity or irregularity of the information of the PFM myoelectrical activity was evaluated by the *SampEn* of the sEMG. In patients, a post-BoNT/A decrement of this feature could initially be expected, since the toxin prevents the development of action potentials in some muscle fibers, which can be interpreted as a reduction of the information generated by motor units. Instead, *SampEn* was found to be higher after the therapy than at the beginning of the study. It has previously been stated that muscular fatigue is characterized by increased synchronization of the motor units together with the aforementioned PSD shift. The hypothesis on the post-BoNT/A recovery from fatigue would thus explain why the complexity (entropy) of the sEMG signal was initially lower in patients than in healthy women and why it rose in the first group after treatment. On the other hand, fibrillatory potentials reported by several authors after the chemical denervation of fiber muscles [36,37] could also be involved in the increase in the *SampEn* value.

In some cases, the aforementioned rising or falling trends in each parameter after BoNT/A infiltration were interrupted in one of the follow-up visits, mainly at Week 12. This could be related to the fact that, subsequent to the denervation produced by BoNT/A, a sprouting of new nerve endings occurs in the muscle to compensate for the function lost by denervated terminals. This growth of new terminals remains for some weeks until the original nerves start restoring their function. Nerve sprouts finally disappear when the original nerves completely recover from the denervation caused by BoNT/A [38]. Considering these events, monotonic changes in the parameters of the sEMG after BoNT/A infiltration should not necessarily occur.

When patients’ sEMG parameters after BoNT/A infiltration were compared with those recorded at the beginning of the study, significant changes were detected in all recording sites. This suggests that part of the neurotoxin probably disseminated to other regions after infiltration. However, the results also point to a greater effect of BoNT/A in the PFM infiltrated and lower sides, whose bipolar recordings showed a greater number of significant differences in their characteristics. In the case of the infiltrated region, this is an expected outcome since BoNT/A was injected precisely there. In the lower side, the reasons could be either that one of its electrodes was closer to the infiltration point than that of the upper side and that gravity also facilitated the downwards diffusion of the toxin. Comparisons between the infiltrated and non-infiltrated sides were not addressed in the present study. However, preliminary analysis showed that only the infiltrated side’s *RMS* was lower than the non-infiltrated side’s *RMS* at Week 12. The absence of a higher number of significant differences could be due to the aforementioned diffusion of the toxin from the infiltrated side towards the opposite side.

According to a review on the assessment of the PFM’s role in sexual pain [39], sEMG is not regarded as a practical tool in daily clinical practice, mainly because a special ability is required to analyze the signal in detail. For this reason, we developed the index of pelvic electrophysiological impairment, a sEMG-based measure that quantifies the alterations present in the patient’s PFM activity from a global perspective. In nature, as the *IPEI* is a distance metric that reflects how far the patient’s PFM electrophysiological condition is from the healthy situation, it can easily be interpreted by clinicians. *IPEI* decreased from the pretreatment situation to the post-treatment visits, and whereas numerous patients’ *IPEI* values were outside the healthy range at the first follow-up visits, almost all the subjects’ *IPEI*s were within it at the end of the study. These results show that the alterations present in the patients’ electrophysiological PFM condition before treatment were mitigated after BoNT/A infiltration. According to the VAS scores, the patients’ painful symptoms also decreased after treatment, showing an agreement between the changes in the patients’ PFM electrophysiological condition and their clinical status.

Significant differences in the patients’ electrophysiological PFM condition before and after BoNT/A infiltration, as well as those in healthy subjects, were found by means of sEMG signals recorded with external adhesive electrodes. This suggests that the activity of the deepest PFM (injected site) significantly contributes to the signal recorded by the electrodes and is not masked by the activity of neighboring muscles or the most superficial PFM bundles. This is an important outcome, since, as opposed to vaginal and anal probes, recording with these electrodes is not an intracavitary technique, which facilitates their acceptance by the patients and their clinical use.

## 5. Conclusions

In this work, relevant changes in the PFM myoelectrical activity of CPP patients were identified after their treatment with BoNT/A: a reduction of its energy, a shift of the spectral content towards higher frequencies and an increase in its information complexity. These changes were more evident during PFM contractions than at rest and lasted at least for the 24 weeks of the follow-up period, when BoNT/A effects were usually maximal. They may be associated with fewer muscle fibers available to develop action potentials, a drop in muscle fatigue and muscle fiber synchronization and the appearance of fibrillatory potentials after BoNT/A chemical denervation.

Whereas BoNT/A was injected at a single point of the PFM, significant changes in the patients’ sEMG were found on all PFM sides, suggesting the diffusion of the toxin. This implies that surface electromyography can also provide useful information on the distribution of BoNT/A in the PFM in treatment for CPP.

The PFM activity of CPP patients was compared with that of healthy women with similar demographic and obstetric characteristics, and a clinically comprehensible metric was developed to quantify the patients’ PFM electrophysiological impairment. This was significant before treatment and decreased after it, becoming insignificant with respect to the healthy condition. This suggests that, apart from the positive effect of BoNT/A on pain management, it brings PFM electrophysiological activity to a state more similar to that of a non-pathological condition.

This study showed the usefulness of recording sEMG by adhesive electrodes to monitor the PFM electrophysiological condition of patients with CPP treated with BoNT/A. Its use could also be extended to assess the effects of other CPP therapies and may also be helpful for the diagnosis of other PFM disorders.

## Figures and Tables

**Table 1:** Clinical characteristics of patients and healthy women.

		Patients	Healthy
Age (years)	mean ± SD	43.8 ± 8.8	40.9 ± 7.2
Weight (kg)	mean ± SD	65.8 ± 11.2	61.8 ± 9.3
Height (cm)	mean ± SD	162.6 ± 5.4	162.0 ± 3.8
Nº deliveries	mean ± SD	1.5 ± 0.7	1.5 ± 0.8
Menopause	Nº subjects (%)	8 (33.3%)	3 (12.5%)
Laterality of pain	Nº subjects (%)	4 (16.6%) Right 10 (41.7%) Left 10 (41.7%) Bilateral	---
Years since pain	mean ± SD	4.8 ± 4.9	---
Initial basal tone	Nº subjects (%)	6 (25%) Normal 2 (8.3%) Hypotonicity 16 (66.7%) Hypertonicity	---

**Table 2:** Parameters computed from sEMG signals of patients and healthy women during PFM contractions. Medians (interquartile ranges) are shown for each PFM side and week of the study (only patients). In healthy women, “Infiltrated” and “Non-infiltrated” rows correspond to the left and right side of the PFM, respectively.

		Week	0	8	12	24	Healthy
*RMS* (µV)	Infiltrated		6.07 (3.13)	4.83 (2.91)	4.61 (3.02)	4.27 (2.39)	2.80 (1.93)
	Non-infiltrated		5.71 (2.57)	4.75 (2.44)	5.74 (2.56)	4.54 (2.94)	2.53 (1.73)
	Upper		7.81 (6.12)	5.86 (5.17)	6.59 (3.16)	5.80 (3.42)	6.96 (4.56)
	Lower		9.45 (4.32)	7.09(3.56)	5.81 (3.73)	6.08 (4.89)	7.67 (3.21)
*MDF* (Hz)	Infiltrated		125 (19)	140 (21)	139 (26)	140 (21)	157 (26)
	Non-infiltrated		134 (32)	135 (21)	132 (18)	138 (34)	136 (26)
	Upper		155 (24)	157 (33)	147 (33)	159 (42)	169 (23)
	Lower		145 (22)	153 (24)	149 (41)	160 (25)	166 (27)
*DI* (10^−14^ Hz^−6^)	Infiltrated		1.63 (1.16)	1.18 (0.61)	1.34 (0.86)	1.14 (0.72)	1.03 (0.89)
	Non-infiltrated		1.36 (1.33)	1.24 (0.83)	1.42 (0.89)	1.04 (0.89)	0.75 (0.57)
	Upper		0.80 (0.92)	0.77 (0.58)	0.95 (0.66)	0.77 (0.63)	0.61 (2.45)
	Lower		1.05(0.51)	0.82 (0.49)	0.79 (0.84)	0.73 (0.31)	0.72 (0.34)
*SampEn*	Infiltrated		0.44 (0.09)	0.46 (0.06)	0.46 (0.08)	0.48 (0.09)	0.50 (0.08)
	Non-infiltrated		0.45 (0.12)	0.45 (0.08)	0.45 (0.09)	0.46 (0.12)	0.55 (0.10)
	Upper		0.51 (0.09)	0.51 (0.07)	0.50 (0.08)	0.51 (0.11)	0.56 (0.05)
	Lower		0.47 (0.07)	0.51 (0.09)	0.50 (0.08)	0.53 (0.04)	0.51 (0.06)

**Table 3:** Parameters computed from sEMG signals of patients and healthy women during PFM relaxation. Medians (interquartile ranges) are shown for each PFM side and week of the study (only patients). In healthy women, “Infiltrated” and “Non-infiltrated” rows correspond to the left and right side of the PFM, respectively.

		Week	0	8	12	24	Healthy
*RMS* (µV)	Infiltrated		1.37 (1.35)	1.32 (1.14)	1.14 (0.74)	1.13 (0.92)	0.95 (0.33)
	Non-infiltrated		1.40 (0.80)	1.37 (0.91)	1.30 (0.86)	1.13 (1.05)	0.94 (0.23)
	Upper		1.62 (0.95)	1.62 (1.49)	1.54 (2.12)	1.37 (1.51)	1.42 (0.55)
	Lower		1.64 (0.74)	1.40 (0.99)	1.33 (0.84)	1.45 (0.89)	1.44 (0.51)
*MDF* (Hz)	Infiltrated		164 (45)	165 (62)	162 (66)	176 (68)	190 (43)
	Non-infiltrated		168 (48)	164 (60)	168 (55)	198 (54)	193 (48)
	Upper		161 (39)	165 (57)	155 (51)	176 (65)	164 (32)
	Lower		146 (43)	165 (32)	168 (44)	164 (39)	171 (30)
*DI* (10^−14^ Hz^−6^)	Infiltrated		0.57 (0.63)	0.56 (0.42)	0.45 (0.80)	0.49 (0.57)	0.36 (0.15)
	Non-infiltrated		0.53 (0.45)	0.56 (0.65)	0.46 (0.64)	0.36 (0.62)	0.33 (0.15)
	Upper		0.61 (0.43)	0.58 (0.80)	0.66 (1.15)	0.43 (1.21)	0.54 (0.25)
	Lower		0.71 (0.39)	0.57 (0.30)	0.53 (0.41)	0.53 (0.33)	0.54 (0.27)
*SampEn*	Infiltrated		0.60 (0.19)	0.68 (0.19)	0.65 (0.20)	0.70 (0.20)	0.70 (0.08)
	Non-infiltrated		0.61 (0.15)	0.65 (0.18)	0.63 (0.18)	0.70 (0.20)	0.72 (0.09)
	Upper		0.61 (0.12)	0.63 (0.16)	0.58 (0.24)	0.61 (0.22)	0.60 (0.10)
	Lower		0.60 (0.09)	0.63 (0.09)	0.62 (0.12)	0.63 (0.09)	0.62 (0.07)

**Table 4:** Significant differences between patients’ sEMG before vs. after BoNT/A. Shadowed cells: rejection of the null hypothesis (Test 1). Arrows: increment (↑) or reduction (↓) in the parameter value with respect to week 0 (one arrow: *p*-value < 0.05; two arrows: *p*-value < 0.01; three arrows: *p*-value < 0.001).

			Contraction			Relaxation		
		Week	8	12	24	8	12	24
*RMS*	Infiltrated		↓↓↓	↓↓↓	↓↓↓	↓		
	Non-infiltrated		↓↓		↓↓			↓
	Upper		↓	↓	↓↓			
	Lower		↓↓↓	↓↓↓	↓↓↓			↓
*MDF*	Infiltrated		↑		↑			↑
	Non-infiltrated				↑			↑
	Upper		↑					
	Lower		↑		↑↑↑	↑	↑	↑
*DI*	Infiltrated		↓↓		↓↓			
	Non-infiltrated		↓		↓↓			
	Upper				↓			
	Lower		↓↓		↓↓↓	↓		↓↓
*SampEn*	Infiltrated		↑	↑	↑↑			
	Non-infiltrated				↑			
	Upper							
	Lower		↑↑		↑↑			↑

**Table 5:** Significant differences between patients vs. healthy women. Shadowed cells: rejection of the null hypothesis (Test 2). Arrows: greater (↑) or lower (↓) parameter value in patients than in healthy women (one arrow: *p*-value < 0.05; two arrows: *p*-value < 0.01; three arrows: *p*-value < 0.001). In healthy women, “Infiltrated” and “Non-infiltrated” rows correspond to the left and right side, respectively.

			Contraction				Relaxation			
		Week	0	8	12	24	0	8	12	24
*RMS*	Infiltrated		↑↑↑	↑		↑	↑↑	↑		
	Non-infiltrated		↑↑↑	↑↑↑	↑↑↑	↑↑	↑↑↑	↑↑	↑↑↑	↑
	Upper									
	Lower				↑					
*MDF*	Infiltrated						↓↓			
	Non-infiltrated		↓↓	↓↓	↓↓↓		↓	↓		
	Upper		↓		↓					
	Lower		↓↓		↓		↓			
*DI*	Infiltrated		↑↑				↑↑	↑		
	Non-infiltrated		↑↑↑	↑↑	↑↑↑		↑	↑↑	↑↑	↑
	Upper		↑	↑	↑↑					
	Lower		↑↑							
*SampEn*	Infiltrated		↓				↓			
	Non-infiltrated		↓↓	↓↓	↓↓	↓	↓↓	↓	↓↓	
	Upper			↓	↓					
	Lower		↓↓							

## Data Availability

The data are not publicly available since subjects enrolled in the study were not explicitly asked whether they consented to the sharing of their data.
